# Does the Type of Anesthetic Technique Affect In-Hospital and One-Year Outcomes after Off-Pump Coronary Arterial Bypass Surgery?

**DOI:** 10.1371/journal.pone.0152060

**Published:** 2016-04-07

**Authors:** Jeong Jin Min, Gahyun Kim, Jong-Hwan Lee, Kwan Young Hong, Wook Sung Kim, Young-Tak Lee

**Affiliations:** 1 Department of Anesthesiology and Pain Medicine, Samsung Medical Center, Sungkyunkwan University School of Medicine, Seoul, Korea; 2 Department of Thoracic and Cardiovascular Surgery, Samsung Medical Center, Sungkyunkwan University School of Medicine, Seoul, Korea; University of Bologna, ITALY

## Abstract

Despite numerous previous studies, there is little data on the effects of anesthetics on clinical outcome after off-pump coronary arterial bypass grafting (OPCAB). Therefore, we retrospectively compared the effects of anesthetic choice on in-hospital major adverse events (MAEs) and one-year major adverse cardiovascular and cerebral events (MACCEs) in patients undergoing OPCAB. Electronic medical records were reviewed in 192 patients who received propofol-remifenanil total intravenous anesthesia (TIVA) and propensity score-matched 662 patients who received isoflurane anesthesia. The primary endpoints were in-hospital MAEs and one-year MACCEs. The components of in-hospital MAEs were in-hospital death, myocardial infarction (MI), coronary revascularization, stroke, renal failure, prolonged mechanical ventilation longer than 72 h, and postoperative new cardiac arrhythmia requiring treatment. One-year MACCEs was defined as a composite of all-cause mortality, MI, coronary revascularization, and stroke. There was no significant difference in risk of in-hospital MAEs (OR = 1.29, 95% CI = 0.88–1.88, *P* = 0.20) or one-year MACCEs (OR = 0.81; 95% CI = 0.46–1.42, *P* = 0.46) between the groups. The risk of postoperative new arrhythmia including new atrial fibrillation significantly increased in the TIVA group compared to the isoflurane anesthesia group (OR = 1.72, 95% CI = 1.12–2.63, *P* = 0.01). In conclusion, the choice between propofol-remifentanil TIVA and isoflurane anesthesia did not show differences in incidence of in-hospital MAEs or one-year MACCEs in patients undergoing OPCAB. However, further studies on the effects of anesthetics on development of in-hospital new arrhythmia will be needed.

## Introduction

In current clinical practice, a volatile agent or propofol-remifentanil are the most frequently chosen anesthetic drugs for cardiac surgeries. Interestingly, all of these agents have been suggested to have cardio-protective effects against ischemia/reperfusion (I/R) injury through different mechanisms. While a pharmacologic preconditioning effect has been considered to be the main mechanism of volatile anesthetics and opioids, propofol has shown antioxidant properties [1, 2]. Moreover, the clinical superiority of one anesthetic technique over another is still controversial [3–9]. Accordingly, numerous studies have investigated the protective effects of anesthetics in cardiac surgeries using cardiopulmonary bypass (CPB), which is accompanied by profound systemic I/R injury [4, 8, 10–13].

Even without the use of CPB, off-pump coronary artery bypass grafting (OPCAB) still exposes patients to surgery-induced myocardial I/R injury. Therefore, the protective effects of anesthetics in OPCAB need to be studied separately from on-pump cardiac surgeries. In addition, considering that the goal of intra-operative care is to improve overall patient outcome, the effects of anesthetics on in- and out-of-hospital complications should be investigated. However, previous research on OPCAB has mainly focused on changes in postoperative cardiac biomarkers and has shown limited clinical outcomes [3, 5, 9, 14, 15].

Therefore, in patients undergoing OPCAB, we compared the effects of two representative anesthetic techniques (isoflurane *versus* propofol-remifentanil total intravenous anesthesia [TIVA]) on in-hospital postoperative major adverse events (MAEs) and one-year major adverse cardiovascular and cerebral events (MACCEs).

## Methods

### Study design and patient population

This study was approved by the Institutional Review Board of Samsung Medical Center (IRB No. 2013-09-127) and was conducted in accordance with the principles of the Declaration of Helsinki. Because this was a retrospective study using electronic medical records, individual informed consent was waived. Patient information was anonymized and de-identified prior to analysis. The study population consisted of adult patients older than 20 years who underwent off-pump coronary arterial bypass grafting between 2010 and 2012 at Samsung Medical Center. Patients were excluded if they required CPB during surgery including elective combined use of CPB or urgent on-pump conversion. For patients who underwent several surgeries, we included only the first surgery in this study.

### Data collection

The electronic medical records of enrolled patients were reviewed, and pre-, intra-, and post-operative data were collected. Laboratory data including serum troponin I, creatine kinase (CK)-MB, creatinine, and N-terminal pro-brain natriuretic peptide (NT-proBNP) levels were extracted automatically from the electronic medical records with the aid of the hospital’s medical informatics department. Postoperative outcome data were collected by manual review of each case by two researchers (J. J. Min and K.Y. Hong) who were blinded to the anesthetic technique.

### Anesthesia technique

Anesthesia was maintained either by propofol with remifentanil or isoflurane inhalation. In the propofol-remifentanil TIVA group, intravenous propofol (1 mg/kg) and a continuous infusion of remifentanil (0.05–0.2 ug/kg/min) were used for anesthesia induction. Anesthesia was maintained with continuous infusion of propofol (80–150 ug/kg/min) and remifentanil (0.05–0.30 ug/kg/min). In the isoflurane group, anesthesia was induced with intravenous etomidate (0.2 mg/kg) and sufentanil (1–2 ug/kg) and then maintained with isoflurane (0.8–1.5 Vol%). The BIS score was monitored and maintained between 40 and 60 in all patients. For neuromuscular blockade, 0.8 mg/kg of rocuronium bromide was used to facilitate tracheal intubation and was maintained with a continuous infusion of vecuronium (8–10 mg/hr) throughout the operation.

### Study endpoints

The primary endpoints were in-hospital MAEs and one-year MACCEs. In-hospital MAEs were a composite of in-hospital death, myocardial infarction (MI), coronary revascularization, stroke, renal failure, prolonged mechanical ventilation longer than 72 h, and new postoperative cardiac arrhythmia requiring treatment. One-year MACCEs were a composite of all-cause mortality, MI, coronary revascularization, and stroke. Definitions of each postoperative outcome are as follows. MI was determined using a new definition of clinically relevant MI after coronary revascularization,[16] and coronary revascularization was confirmed through review of hospital records. Stroke was defined as a new ischemic or hemorrhagic cerebrovascular accident with a neurological deficit lasting longer than 24 h. Renal failure was defined as an increase in serum creatinine > 2.0 and more than two times the most recent preoperative creatinine level or a new requirement for postoperative dialysis [17]. New cardiac arrhythmia requiring treatment included new postoperative atrial fibrillation or potentially fatal ventricular arrhythmia requiring immediate treatment. Postoperative wound problem was defined as any sternal wound complication after surgery including mediastinitis.

### Statistical analysis

Preoperative characteristics such as patient comorbidities or number of diseased coronary arteries might bias the choice of anesthetic technique. To eliminate this bias, patients receiving TIVA were matched with those receiving isoflurane anesthesia based on propensity score. Because isoflurane anesthesia was used more frequently than propofol-remifentanil TIVA for our OPCAB patients during the study period, we used 1:N matching rather than 1:1 matching so as to minimize the loss of control subjects. A previous study has reported that 1:N matching method is superior to 1:1 matching in terms of efficiency without reducing precision [18]. Logistic regression was used to calculate exposure propensity scores of likelihood of receiving TIVA using all variables listed in Table 1. After propensity score matching, the balance between the two groups was evaluated using standardized mean difference, variance ratio, and overall distributions. A standardized difference less than 10% was considered a good balance between groups.

**Table 1:** Characteristics of matched variables, before and after propensity score matching.

		Before matching					After propensity score matching				
		TIVA		Isoflurane		STD*	TIVA		Isoflurane		STD*
		(n = 195)		(n = 720)			(n = 192)		(n = 662)		
		No.	(%)	No.	(%)		No.	(%)	No.	(%)	
Age, yr		63.75 ± 9.43		63.64 ± 9.00		1.12	63.64 ± 9.43		63.81 ± 9.12		1.89
Sex, Male		144	(74)	570	(79)	12.08	142	(74)	494	(75)	1.67
Current smoker		34	(17)	128	(18)	0.9	34	(18)	117	(18)	0.09
Body mass index, kg/m^2^		24.05 ± 3.07		24.51 ± 3.00		15.14	24.09 ± 3.08		24.13 ± 2.88		1.58
EuroSCORE		4.09 ± 2.66		4.10 ± 2.49		0.59	4.07 ± 2.66		4.14 ± 2.49		2.58
Comorbidities											
	Hypertension	131	(67)	481	(67)	0.79	128	(67)	432	(65)	2.91
	Diabetes Mellitus or HbA1C >6.5%	104	(53)	397	(55)	3.61	103	(54)	337	(51)	5.61
	Dyslipidemia	48	(25)	202	(28)	7.97	47	(24)	173	(26)	3.7
	History of old MI	5	(3)	23	(3)	3.98	5	(3)	16	(2)	1.04
	Previous PCI	33	(17)	117	(16)	1.79	33	(17)	106	(16)	3.07
	Peripheral vascular disease	15	(8)	53	(7)	1.24	15	(8)	52	(8)	0.1
	COPD	1	(1)	3	(0)	1.34	1	(1)	3	(0)	1.21
	History of stroke	16	(8)	94	(13)	17.63	16	(80	60	(9)	2.9
	Chronic liver disease	0	(0)	9	(1)	0	0	(1)	0	(1)	0
	Chronic kidney disease	7	(4)	39	(5)	9.8	7	(4)	24	(4)	0
MI within 4 weeks or UA within 8 weeks		93	(48)	365	(51)	6	92	(48)	324	(49)	1.99
Preoperative LV EF (%)		57.45 ± 11.12		57.61 ± 11.46		1.43	57.55 ± 11.17		57.31 ± 11.49		2.69
Preoperative NT-proBNP		657.97 ± 1671.92		605.85 ± 2282.27		8.82	655.71 ± 1683.88		651.75 ± 2290.25		1.6
Medication											
	Angiotensin converting enzyme inhibitor	21	(11)	86	(12)	3.78	21	(11)	76	(12)	1.84
	Angiotenson receptor blocker	56	(29)	173	(24)	10.34	53	(28)	169	(26)	4.57
	Aspirin	128	(66)	488	(68)	4.49	126	(66)	421	(64)	4.19
	Beta blocker	60	(31)	203	(28)	5.56	57	(30)	197	(30)	0.23
	Clopidogrel	86	(44)	316	(44)	0.43	85	(44)	289	(44)	1.1
	Diuretics	36	(18)	114	(16)	6.76	33	(17)	109	(64)	2.01
	Insulin	8	(4)	42	(6)	8.7	8	(4)	26	(4)	1.31
	Oral hypoglycemic agents	56	(29)	220	(31)	4.05	55	(29)	178	(27)	3.88
	Statin	85	(44)	324	(45)	2.84	84	(44)	287	(43)	0.75
Intraoperative data											
	Redo operation	0	(0)	8	(1)	0	0	(0)	0	(0)	0
	Three vessel disease	147	(75)	507	(70)	11.5	144	(75)	493	(74)	1.19
	Left main disease	41	(21)	173	(24)	7.35	41	(21)	147	(22)	2.25
	Emergent operation	36	(18)	130	(18)	1.04	35	(18)	116	(18)	1.7
	Number of distal grafts	4.05 ± 1.24		4.08 ± 1.35		2.59	4.04 ± 1.22		4.03 ± 1.33		0.83
	Vein graft	27	(14)	93	(13)	2.68	27	(14)	96	(15)	1.2
	Duration of surgery, min	346.56 ± 78.99		337.88 ± 71.55		9.68	345.25 ± 78.10		343.23 ± 72.20		1
	Number of transfused packed RBCs, u	1.80 ± 1.52		1.85 ± 1.63		3.64	1.79 ± 1.51		1.77 ± 1.54		1.37
	Number of used inotropics or vasopressor	1.74 ± 0.89		1.68 ± 0.88		6.19	1.73 ± 0.89		1.71 ± 0.89		2.84
	Perioperative IABP	1	(1)	4	(1)	0.6	1	(1)	3	(0)	0.97
	Fatal ventricular arrhythmia	2	(1)	5	(1)	3.28	2	(1)	4	(1)	4.3

TIVA indicates propofol-remifentanil total intravenous anesthesia; STD, standardized difference; MI, myocardial infarction; PCI, percutaneous coronary intervention; COPD, chronic obstructive pulmonary disease; UA, unstable angina; LV EF, left ventricular ejection fraction; NT-proBNP, N-terminal pro-brain natriuretic peptide; RBCs, red blood cells; IABP, intra-aortic balloon pump.

* Standardized difference (STD) of greater than 10 percent represented meaningful imbalance between study groups.

Continuous variables are presented as mean ± SD, and categorical variables as number and percentage. Student’s *t*-test or Mann-Whitney test was used to compare pre- and post-matched continuous covariates of patients or surgical characteristics between the groups, and the Chi-square test was used for categorical variables as appropriate. Some continuous variables were log-transformed to make them closer to symmetric normal distributions (e.g., duration of surgery; laboratory values of pre- and postoperative NT-proBNP, troponin I, and CKMB; postoperative duration of mechanical ventilation; and length of ICU stay). To estimate the odds ratio (OR) and 95% confidence interval (CI) for risk of dichotomous in-hospital postoperative outcome according to TIVA, we used the Generalized Estimation Equations (GEE) method. For comparison of in-hospital continuous outcomes (e.g., postoperative laboratory data or length of stay), we used GEE to perform weighted linear regression with cluster analysis. A matched Cox proportional hazard model identified the risk factors of one-year MACCEs according to TIVA exposure. We verified the proportional hazard assumptions.

Subgroup analyses were conducted to further clarify our results. We estimated the risks of study outcomes in patients with or without higher risk (older than 70 yr and left ventricular ejection fraction lower than 50%) or with diabetes mellitus or high HbA_1_C. All statistical analysis analyses were performed using the Statistical Analysis System (release 9.3; SAS Institute, Inc., Cary, NC, USA).

## Results

### Patient and surgical characteristics

**Fig 1:**
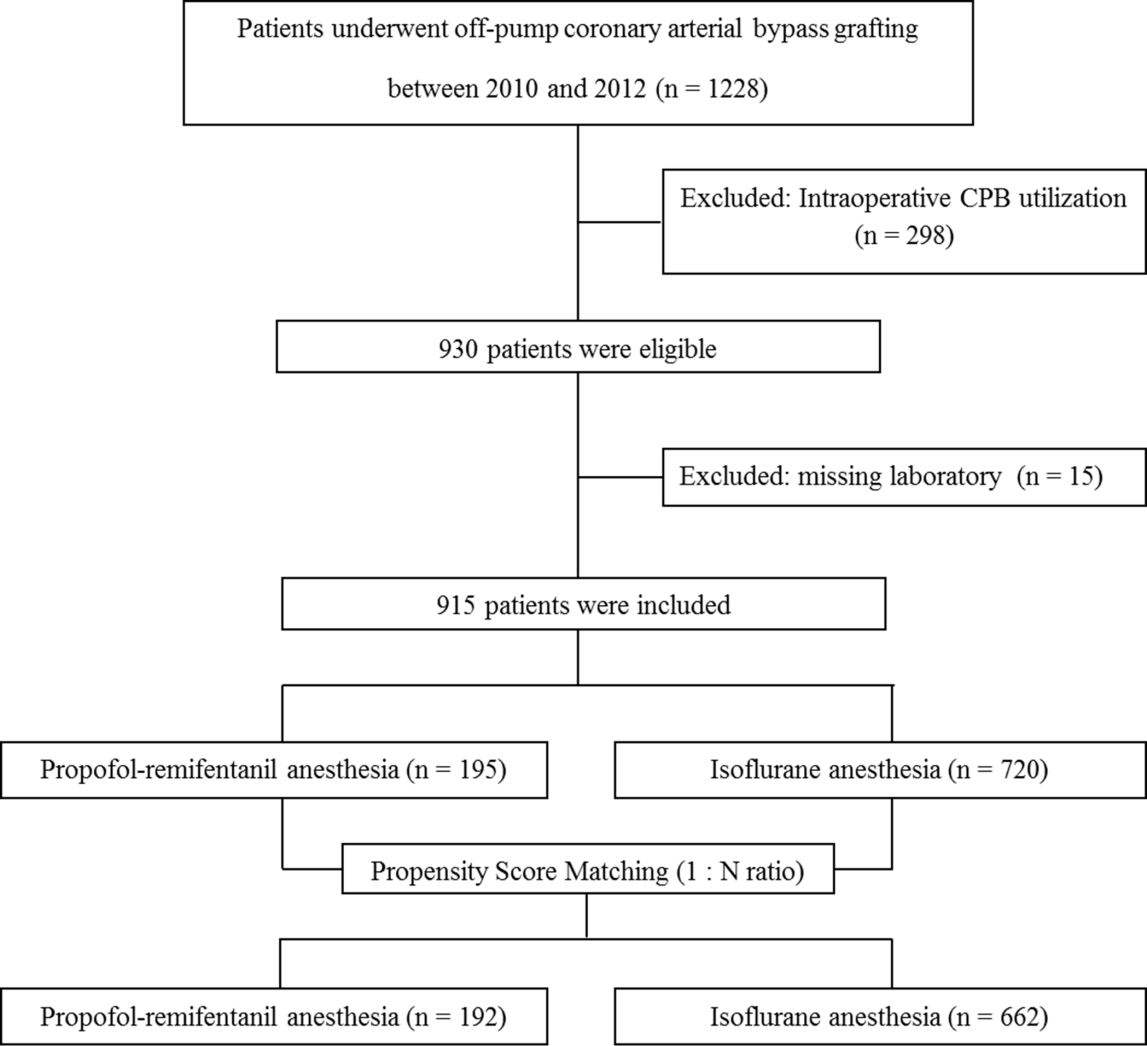
Flow diagram outlining the selection of study population.

A total of 1228 patients who underwent OPCAB during the study periods were screened and 298 patients who required intraoperative CPB were excluded (Fig 1). Among the remaining 930 patients, we enrolled 915 patients for which the values of all variables for propensity score matching were available (Fig 1). After one-to-many matching according to propensity score, 192 patients who received TIVA were matched with 662 patients who received isoflurane anesthesia, for a total of 854 patients (Fig 1). The patient, surgical, and laboratory characteristics before and after matching are listed in Table 1. The two groups contained some mismatched baseline characteristics including sex, body mass index (BMI), previous stroke, presence of three-vessel disease, and usage of angiotensin receptor blocker before propensity score matching (standardized differences > 10%); however, there were no significant differences in any variables between the study groups in the propensity score-matched cohort (Table 1).

### In-hospital MAEs and anesthetic techniques

The incidences of in-hospital MAEs and its components according to anesthetic method are shown in Table 2. The rate of in-hospital MAEs was 26% (49/192) in the TIVA group and 21% (139/662) in the isoflurane group, and there was no significant difference between the groups (OR = 1.29, 95% CI = 0.88–1.88, *P* = 0.20). In addition, the risk of any component of in-hospital MAEs did not differ between the two anesthetic techniques except postoperative development of a new arrhythmia (Table 2). The risk of postoperative new arrhythmia including new atrial fibrillation significantly increased in the TIVA group compared to the isoflurane anesthesia group (OR = 1.72, 95% CI = 1.12–2.63, *P* = 0.01 for postoperative new arrhythmia and OR = 1.58, 95% CI = 1.01–2.45, *P* = 0.04 for new atrial fibrillation, Table 2). There was one in-hospital postoperative death in the isoflurane anesthesia group. It was not possible to estimate the odds ratio of in-hospital death.

**Table 2:** Risks of postoperative complications according to the anesthetic method based on matched data.

		TIVA	Isoflurane	OR or HR	95% CI	*P*-value
		(n = 192)	(n = 662)			
Composite of in-hospital MAEs		49 (25.5)	139 (21)	1.29	0.88–1.88	0.2
	In-hospital death	0 (0)	1 (0.2)			
	In-hospital myocardial infarction	11 (5.7)	47 (7.1)	0.8	0.41–1.57	0.52
	In-hospital revascularization	1 (0.5)	4 (0.6)	0.86	0.09–7.79	0.89
	In-hospital stroke	2 (1)	7 (1.1)	0.99	0.21–4.78	0.99
	Prolonged mechanical ventilation (>72h)	23 (12)	67 (10.1)	0.84	0.18–3.98	0.83
	Acute kidney injury	5 (2.6)	24 (3.6)	0.7	0.25–1.93	0.49
	In-hospital new arrhythmia	38 (19.8)	82 (12.4)	1.72	1.12–2.63	0.01
	New atrial fibrillation	32 (16.7)	74 (11.2)	1.58	1.01–2.45	0.04
	Postoperative ventricular arrhythmia	6 (3.1)	8 (1.2)	2.55	0.96–6.75	0.06
One year MACCEs		14 (7.3)	58 (8.8)	0.81	0.46–1.42	0.46
	Death	1 (0.5)	2 (0.3)	2.22	0.20–25.05	0.52
	Myocardial infarction	11 (6)	44 (6.6)	0.8	0.42–1.53	0.51
	Revascularization	1 (0.5)	9 (1.4)	0.39	0.05–3.10	0.37
	Stroke	2 (1)	9 (1.4)	0.89	0.19–4.18	0.89
Other postoperative outcomes						
	Prolonged ICU stay (>72h)	23 (12)	67 (10.1)	0.83	0.51–1.35	0.45
	In-hospital wound problem	5 (2.6)	15 (2.3)	1.14	0.39–3.30	0.81
	Bleeding-related reoperation	4 (2)	7 (1.1)	1.96	0.57–6.68	0.28
	Time to extubation, hr	8 [6–12]	8 [6–12]			0.71*
	Length of stay at ICU, hr	34.5 [20–45.63]	35.75 [22–48]			0.49*
	CKMB_max_	9.94 [6.79–15.65]	10.9 [7.01–16.89]			0.44*
	Troponin I_max_	2.29 [1.33–4.73]	2.70 [1.32–5.07]			0.68*
	NT-proBNP_max_	361.3 [155–711.9]	346.97 [154.57–713.91]			0.26*

Data are presented as number (%) or median [interquartile range].

*P-value was analyzed with the log-transformed data.

TIVA indicates propofol-remifentanil total intravenous anesthesia; OR, odds ratio; HR, hazard ratio; CI, confidence interval; MAEs, major adverse events; MACCEs, major adverse cardiovascular and cerebral events; ICU, intensive care unit; CKMB_max_, postoperative maximum creatine kinase-MB; NT-proBNP_max_, postoperative maximum N-terminal pro-brain natriuretic peptide.

### One-year MACCEs and anesthetic techniques

During the follow-up period (up to one year postoperatively), postoperative MACCEs occurred in 8.4% (72/854) of all patients. Postoperative MACCEs included 3 deaths, 55 MIs, 10 coronary revascularizations, and 11 strokes. The incidence of one-year MACCEs was 7.3% (14/192) in the TIVA group and 8.8% (58/662) in the isoflurane anesthesia group. There was no significant differences in occurrence of MACCEs between the groups (OR = 0.81, 95% CI = 0.46–1.42, *P* = 0.46), including any of its components (Table 2).

### Other postoperative outcomes

The incidences of prolonged ICU stay (>72 hr), postoperative wound problem, need for bleeding-related reoperation, time to extubation, length of ICU stay, and postoperative maximal values of serum CKMB, troponin I, and NT-proBNP did not differ between the two groups (Table 2). The values of postoperative serum troponin I and NT-proBNP were available only in 712 and 822 patients, respectively.

### Subgroup analyses

Because volatile agents have showed a superior protective effect compared to propofol on postoperative myocardial damage in elderly high-risk coronary surgery patients with impaired myocardial function, although these two approaches have been comparable in studies of patients with good cardiac function [13], additional analyses were performed in high-risk patients (n = 352). However, neither in-hospital MAEs nor one-year MACCEs showed a significant difference between the two anesthetic methods (Table 3). Moreover, the presence of diabetes mellitus and hyperglycemia and the use of oral hypoglycemic drugs have been reported to attenuate the beneficial effects of preconditioning [2, 19, 20]. Therefore, we also performed subgroup analyses in this population (n = 501). In this subgroup analyses, neither in-hospital MAEs nor one-year MACCEs showed significant differences between the two groups (Table 3).

**Table 3:** Subgroup analyses for risks of postoperative complications according to the anesthetic method based on original data.

			TIVA, n (%)	Isoflurane, n (%)	OR or HR	95% CI	*P*-value
**Subgroup with high-risk (age≥70 or LV EF <45%)**			70	282			
	Composite of in-hospital MAEs		21 (30)	73 (25.9)	1.23	0.69–2.18	0.49
		In-hospital death	0 (0)	0 (0)			
		In-hospital myocardial infarction	3 (4.3)	19 (6.7)	0.62	0.18–2.16	0.45
		In-hospital revascularization	0 (0)	3 (1)			
		In-hospital stroke	1 (1.4)	3 (1)	1.35	0.14–13.16	0.8
		Prolonged mechanical ventilation (>72h)	0 (0)	6 (2.1)			
		Acute kidney injury	3 (4.3)	15 (5.3)	0.8	0.22–2.83	0.73
		In-hospital new arrhythmia	17 (24.3)	49 (17.4)	1.53	0.82–2.86	0.19
	One year MACCEs		5 (7.1)	23 (8.2)	1.54	0.23–1.86	0.42
		Death	1 (1.4)	1 (0.4)			1
		Myocardial infarction	3 (4.3)	17 (6)	1.63	0.18–2.06	0.43
		Revascularization	0	3 (1)			
		Stroke	1 (1.4)	5 (1.8)	1.01	0.11–8.83	0.99
	Other postoperative outcomes						
		Prolonged ICU stay (>72h)	14 (20)	38 (13.5)	1.61	0.82–3.16	0.17
		In-hospital wound problem	2 (2.9)	8 (2.8)	1.01	0.21–4.85	0.99
		Bleeding-related reoperation	2 (2.9)	3 (1.1)	2.74	0.45–16.69	0.28
**Subgroup without high-risk (age<70 or LV EF ≥45%)**			125	438			
	Composite of in-hospital MAEs		28 (22.4)	80 (18.3)	1.29	0.78–2.09	0.3
		In-hospital death	0 (0)	1 (0.2)			
		In-hospital myocardial infarction	8 (6.4)	26 (5.9)	1.08	0.48–2.46	0.85
		In-hospital revascularization	1 (0.8)	2 (0.5)	1.76	0.16–19.55	0.65
		In-hospital stroke	1 (0.8)	5 (1.1)	0.7	0.08–6.03	0.74
		Prolonged mechanical ventilation (>72h)	2 (1.6)	5 (1.1)	1.41	0.27–7.35	0.68
		Acute kidney injury	2 (1.6)	12 (2.7)	0.58	0.13–2.61	0.48
		In-hospital new arrhythmia	20 (16)	45 (10.3)	1.66	0.94–2.94	0.08
	One year MACCEs		10 (8)	36 (8.2)	0.98	0.49–1.98	0.96
		Death	1 (0.8)	1 (0.2)	3.54	0.22–56.52	0.37
		Myocardial infarction	8 (6.4)	26 (5.9)	1.09	0.49–2.41	0.83
		Revascularization	1 (0.8)	6 (1.4)	0.58	0.07–4.85	0.62
		Stroke	1 (0.8)	5 (1.1)	0.7	0.08–6.01	0.75
	Other postoperative outcomes						
		Prolonged ICU stay (>72h)	9 (7.2)	31 (7.1)	1.02	0.47–2.20	0.96
		In-hospital wound problem	3 (2.4)	8 (1.8)	1.32	0.35–5.06	0.68
		Bleeding-related reoperation	2 (1.6)	7 (1.6)	1	0.21–4.88	0.99
**Subgroup with diabetes mellitus or hyperglycemia**			104	397			
	Composite of in-hospital MAEs		27 (26)	92 (23.2)	1.16	0.71–1.91	0.55
		In-hospital death	0 (0)	0 (0)			
		In-hospital myocardial infarction	4 (3.9)	23 (5.8)	0.65	0.22–1.92	0.44
		In-hospital revascularization	1 (1)	3 (0.8)	1.28	0.13–12.39	0.83
		In-hospital stroke	1 (1)	7 (1.8)	0.54	0.07–4.45	0.57
		Prolonged mechanical ventilation (>72h)	2 (1.9)	7 (1.8)	1.09	0.22–5.34	0.91
		Acute kidney injury	3 (2.9)	19 (4.8)	0.59	0.17–2.04	0.4
		In-hospital new arrhythmia	22 (21.1)	59 (14.9)	1.54	0.89–2.65	0.12
	One year MACCEs		6 (5.8)	32 (8.1)	1.4	0.3–1.71	0.45
		Death	1 (1)	2 (0.5)	0.51	0.18–21.64	0.58
		Myocardial infarction	4 (3.9)	23 (5.8)	1.5	0.2–1.9	0.46
		Revascularization	1 (1)	4 (1)	1.05	0.11–8.52	0.97
		Stroke	1 (1)	8 (2)	2.13	0.06–3.76	0.48
	Other postoperative outcomes						
		Prolonged ICU stay (>72h)	17 (16.4)	43 (10.8)	1.61	0.88–2.96	0.13
		In-hospital wound problem	3 (2.9)	10 (2.5)	1.15	0.31–4.26	0.83
		Bleeding-related reoperation	2 (1.9)	7 (1.8)	1.09	0.22–5.34	0.91
**Subgroup without diabetes mellitus or hyperglycemia**			91	323			
	Composite of in-hospital MAEs		22 (24.2)	61 (18.9)	1.37	0.79–2.39	0.27
		In-hospital death	0 (0)	0 (0)			
		In-hospital myocardial infarction	7 (7.7)	22 (6.8)	1.14	0.47–2.76	0.77
		In-hospital revascularization	0 (0)	2 (0.6)			
		In-hospital stroke	1 (1.1)	1 (0.3)	3.58	0.22–57.77	0.37
		Prolonged mechanical ventilation (>72h)	0 (0)	4 (1.2)			
		Acute kidney injury	2 (2.2)	8 (2.5)	0.89	0.19–4.24	0.88
		In-hospital new arrhythmia	15 (16.5)	35 (10.8)	1.62	0.84–3.13	0.15
	One year MACCEs		8	29	0.99	0.46–2.18	0.99
		Death	0 (0)	0 (0)			
		Myocardial infarction	7 (7.7)	23 (7.1)	1.1	0.47–2.57	0.82
		Revascularization	0 (0)	6 (1.9)			
		Stroke	1 (1.1)	1 (0.3)	3.58	0.22–57.29	0.37
	Other postoperative outcomes						
		Prolonged ICU stay (>72h)	6 (6.6)	26 (8.05)	0.81	0.32–2.02	0.65
		In-hospital wound problem	2 (2.2)	6 (1.9)	1.19	0.24–5.98	0.84
		Bleeding-related reoperation	2 (2.2)	3 (0.9)	2.4	0.39–14.57	0.34

TIVA indicates propofol-remifentanil total intravenous anesthesia; OR, odds ratio; HR, hazard ratio; CI, confidence interval; LV EF, left ventricular ejection fraction; MAEs, major adverse events; MACCEs, major adverse cardiovascular and cerebral events; ICU, intensive care unit.

### Postoperative atrial fibrillation and patient outcome

New postoperative in-hospital atrial fibrillation occurred more frequently in the TIVA group than in the isoflurane anesthesia group. Because development of postoperative atrial fibrillation has been reported to be associated with adverse postoperative outcome [21, 22], we additionally analyzed the perioperative risk factors of atrial fibrillation and the effect of atrial fibrillation on postoperative patient outcome. Among the various perioperative variables, older age, higher EuroSCORE, preoperative LV EF < 45%, use of oral hypoglycemic agents, increased number of intraoperative vasoactive drugs, and red blood cells transfusion resulted in an increased risk of postoperative new atrial fibrillation (S1 Table). Patients with new postoperative atrial fibrillation showed higher postoperative maximum troponin I and CK-MB levels, mechanical ventilation time longer than 72 h, ICU stay, and increased risk of in-hospital MI and stroke (Table 4). With regard to long-term outcome, postoperative atrial fibrillation increased the occurrence of one-year MACCEs and its components including PMI, stroke, and death (Table 4).

**Table 4:** Risks of postoperative complications in patients with new postoperative atrial fibrillation.

		OR or HR	95% CI	*P*-value
Composite of in-hospital MAEs				
	In-hospital death			
	In-hospital myocardial infarction	4.08	2.18–7.61	<0.0001
	In-hospital revascularization			
	In-hospital stroke	4.1	0.99–16.88	0.051
	Prolonged mechanical ventilation (>72h)	15.17	4.31–53.46	<0.0001
	Acute kidney injury	1.77	0.73–4.28	0.21
One year MACCEs		2.93	1.76–4.87	<0.001
	Death	11.91	1.06–133.32	0.04
	Myocardial infarction	3.56	2.06–6.28	<0.0001
	Revascularization			
	Stroke	4.7	1.29–17.15	0.02
Other postoperative outcomes				
	Prolonged ICU stay (>72h)	2.35	1.36–4.06	0.002
	In-hospital wound problem	1.6	0.52–4.97	0.41
	Bleeding-related reoperation	2.66	0.76–9.35	0.13
	Time to extubation, hr			0.013
	Length of stay at ICU, hr			<0.001
	CKMB_max_			<0.001
	Troponin I_max_			0.001
	NT-proBNP_max_			0.07

OR indicates odds ratio; HR, hazard ratio; CI, confidence interval; MAEs, major adverse events; MACCEs, major adverse cardiovascular and cerebral events; ICU, intensive care unit;; CKMB_max_, postoperative maximum creatine kinase-MB; NT-proBNP_max_, postoperative maximum N-terminal pro-brain natriuretic peptide.

## Discussion

This propensity score-matched retrospective cohort study showed that the choice of anesthetic (propofol-remifentanil TIVA *versus* isoflurane anesthesia) was not associated with incidence of in-hospital MAEs or one-year MACCEs in patients undergoing OPCAB. This result was consistent in all analyses of the overall and subgroup populations (old age, high risk subgroup and diabetic-hyperglycemic subgroup). However, the incidence of in-hospital postoperative new arrhythmia including atrial fibrillation was increased in the propofol-remifentanil TIVA group.

The protective effects of volatile anesthesia and propofol-based TIVA against I/R injury have been compared in numerous studies because different anesthetics appear to have different protective mechanisms [1]. However, most previous studies have focused on the changes in cardiac biomarkers during the early postoperative period; therefore, there is limited data on the effects of anesthetics on the various clinical outcomes [4, 5, 8, 9, 13–15]. Therefore, it is still difficult to conclude whether one anesthetic approach is superior to the other in terms of patient outcome.

A recent meta-analysis has reported that volatile anesthesia was more effective in reducing postoperative mortality than TIVA, which would be inconsistent with our results [12]. However, this meta-analysis was primarily based on studies of all cardiac surgeries with or without the use of CPB. In addition, we believe that those previous results might have been largely influenced by a single study that showed a much higher one-year mortality rate than what has previously been reported (12.4% in the TIVA group and 4.8% in the desflurane or sevoflurane group) [23]. Moreover, although many previous studies have suggested that volatile anesthetic agents have superior myocardial protective effects to propofol [7, 8, 11, 12, 14, 24], the superiority of volatile anesthetics for clinical outcomes such as length of stay in ICU or hospital did not show consistent results [6, 8, 15]. Furthermore, there have been several studies that have reported that propofol appears to be better than volatile agents in terms of renal [25, 26] or cerebral [10] protection.

In OPCAB studies, the results of several small-sized clinical trials have also been inconsistent with ours [9, 14, 15, 27, 28]. However, because the sample sizes in these studies were calculated based on the changes in cardiac biomarkers or myocardial performance index, the power did not seem to be sufficient to detect differences in clinical outcomes [9, 14, 15, 27, 28]. In addition, the real clinical effects of these reported differences in troponin level with different anesthetics were not clarified in some studies [6, 8, 13, 15]. Moreover, two previous studies of OPCAB patients have demonstrated no differences in myocardial injury markers or long-term outcome between volatile anesthetics and propofol groups [3, 5]. Two recent studies on cardiac surgeries using CPB have also shown no beneficial effect of volatile anesthetics on clinical outcome [4, 29].

Unexpectedly, the incidence of postoperative cardiac arrhythmia, specifically the risk of new atrial fibrillation, was higher in the TIVA group than in the isoflurane anesthesia group in our study. The development of postoperative atrial fibrillation has been associated with higher early morbidity and long-term mortality rate after off- and on-pump coronary bypass surgery, although this is mostly a self-limiting condition [21, 22]. Consistent with these results, our subgroup experiencing postoperative new atrial fibrillation had increased risk of in-hospital MI, prolonged time on mechanical ventilation, longer length of ICU stay, and more one-year MACCEs. The generally accepted clinical risk factors for developing postoperative atrial fibrillation (e.g. preoperative history of cardiac failure, high EuroSCORE, advanced age, male sex, and presence of hypertension) [21, 22, 30] were all matched between the two groups. Therefore, our result is difficult to explain; however, in reality, little is known about the effect of anesthetic method on development of postoperative atrial fibrillation. Considering the relationships between new postoperative atrial fibrillation and short- and long-term clinical outcomes, well-controlled prospective studies examining this issue are necessary.

This retrospective study had two limitations. First, our study was not a randomized prospective study; therefore, there is the possibility of hidden bias from the confounding factors excluded from propensity scoring. Second, in this retrospective study, it was impossible to compare the effect of isoflurane directly to that of propofol because remifentanil was only used in the TIVA group. In addition, the respective contribution of each TIVA drug to our results could not be distinguished because accurate plasma concentration of each drug was not available. Therefore, it was impossible to conclude whether our results were caused by differences in cardioprotective effects between isoflurane and propofol. However, in general clinical practice, continuous infusion of propofol is almost always used in conjunction with continuous opioids, whereas volatile agents are frequently used alone. Therefore, we believe that our comparison is clinically relevant and worthwhile.

In conclusion, the risk of composite in-hospital adverse outcomes or one-year MACCEs was not different between patients receiving isoflurane anesthesia or propofol-remifentanil TIVA. Therefore, these two representative anesthetic techniques would be both acceptable in patients undergoing OPCAB. However, further well-controlled studies will be needed to elucidate the effects of anesthetics on the development of new postoperative atrial fibrillation.

## Supporting Information

S1 Table Risk factors for postoperative new atrial fibrillation. (DOCX)
