# Supplementary material for: Does the Type of Anesthetic Technique Affect In-Hospital and One-Year Outcomes after Off-Pump Coronary Arterial Bypass Surgery?
Source: PLoS One. 2016 Apr 7;11(4):e0152060. doi: 10.1371/journal.pone.0152060 (PMC4824512; doi:10.1371/journal.pone.0152060)
Supplement: S1 Table — (DOCX) [file pone.0152060.s001.docx]

**Supporting Information**

**S1 Table**. Risk factors for postoperative new atrial fibrillation

|  |  | OR | 95% CI | *P*-value |
| --- | --- | --- | --- | --- |
| Age, yr | | 1.05 | 1.02-1.07 | <0.001 |
| Sex, Male | | 1.03 | 0.65-1.63 | 0.9 |
| Current smoker | | 0.69 | 0.4-1.21 | 0.2 |
| Body mass index, kg/m^2^ | | 0.98 | 0.92-1.05 | 0.62 |
| EuroSCORE | | 1.12 | 1.04-1.21 | 0.003 |
| Comorbidities | |  |  |  |
|  | Hypertension | 1.16 | 0.77-1.77 | 0.48 |
|  | Diabetes Mellitus | 1.24 | 0.84 | 1.82 |
|  | Dyslipidemia | 0.76 | 0.48-1.2 | 0.24 |
|  | History of old MI | 1.13 | 0.39-3.33 | 0.82 |
|  | Previous PCI | 1.1 | 0.66-1.84 | 0.7 |
|  | Peripheral vascular disease | 1.19 | 0.59-2.4 | 0.63 |
|  | History of stroke | 0.66 | 0.33-1.3 | 0.23 |
|  | Chronic liver disease | 0.9 | 0.11-7.35 | 0.93 |
|  | Chronic kidney disease | 1.24 | 0.54-2.86 | 0.61 |
| MI within 4 weeks or UA within 8 weeks | | 1.11 | 0.75-1.63 | 0.6 |
| Preoperative LV EF (%) | | 0.99 | 0.97-1 | 0.17 |
| Preoperative NT-proBNP | | 1 |  | 0.08 |
| Medication | |  |  |  |
|  | Angiotensin converting enzyme inhibitor | 1.11 | 0.62-1.99 | 0.72 |
|  | Angiotenson receptor blocker | 1.27 | 0.83-1.95 | 0.27 |
|  | Aspirin | 1.09 | 0.72-1.66 | 0.67 |
|  | Beta blocker | 1.47 | 0.98-2.21 | 0.06 |
|  | Clopidogrel | 1.16 | 0.79-1.7 | 0.46 |
|  | Diuretics | 0.76 | 0.43-1.33 | 0.33 |
|  | Insulin | 1.54 | 0.73-3.27 | 0.26 |
|  | Oral hypoglycemic agents | 1.7 | 1.14-2.53 | 0.009 |
|  | Statin | 1.47 | 0.99-2.16 | 0.05 |
| Intraoperative data | |  |  |  |
|  | Redo operation | 1.04 | 0.13-8.55 | 0.97 |
|  | Three vessel disease | 0.85 | 0.56-1.3 | 0.46 |
|  | Left main disease |  |  |  |
|  | Emergent operation | 1.09 | 0.67-1.78 | 0.72 |
|  | Number of distal grafts | 1.11 | 0.95-1.29 | 0.16 |
|  | Vein graft | 2.08 | 1.28-3.39 | 0.003 |
|  | Duration of surgery, min | 1 | 0.99-1 | 0.72 |
|  | Number of transfused packed RBCs, u | 1.18 | 1.05-1.32 | 0.004 |
|  | Number of used inotropics or Vasopressor | 1.4 | 1.13-1.73 | 0.002 |
|  | Perioperative IABP | 1.69 | 0.19-15.31 | 0.64 |
|  | Fatal ventricular arrhythmia | 1.09 | 0.13-9.13 | 0.94 |

OR indicates odds ratio; CI, confidence interval; MI, myocardial infarction; PCI, percutaneous coronary intervention; UA, unstable angina; LV EF, left ventricular ejection fraction; NT-proBNP, N-terminal pro-brain natriuretic peptide; RBCs, red blood cells; IABP, intra-aortic balloon pump.
